# Paratuberculosis: The Hidden Killer of Small Ruminants

**DOI:** 10.3390/ani12010012

**Published:** 2021-12-21

**Authors:** Sanaa M. Idris, Kamal H. Eltom, Julius B. Okuni, Lonzy Ojok, Wisal A. Elmagzoub, Ahmed Abd El Wahed, ElSagad Eltayeb, Ahmed A. Gameel

**Affiliations:** 1 Department of Animal Health and Safety of Animal Products, Institute for Studies and Promotion of Animal Exports, University of Khartoum, Shambat 13314, Khartoum North, Sudan; sanaaidris15@gmail.com (S.M.I.); wisalelmagzoub@gmail.com (W.A.E.); 2 Department of Pathology, Faculty of Veterinary Medicine, University of Khartoum, Shambat 13314, Khartoum North, Sudan; aargameel@hotmail.com; 3 College of Veterinary Medicine, Animal Resources and Biosecurity (COVAB), Makerere University, Kampala P.O. Box 7062, Uganda; jbokuni@gmail.com (J.B.O.); lonzyo@yahoo.com (L.O.); 4 Department of Pathology, Faculty of Medicine, Gulu University, Gulu P.O. Box 166, Uganda; 5 Department of Biology and Biotechnology, College of Applied and Industrial Sciences, University of Bahri, Alkadaro 13311, Khartoum North, Sudan; 6 Institute of Animal Hygiene and Veterinary Public Health, Faculty of Veterinary Medicine, University of Leipzig, An den, Tierkliniken 43, D-04103 Leipzig, Germany; 7 Faculty of Medicine, Al Neelain University, Almogran 11111, Khartoum, Sudan; sagadgady@yahoo.com

**Keywords:** paratuberculosis, small ruminants, neglected disease

## Abstract

**Simple Summary:**

Paratuberculosis is a chronic disease of ruminants and many non-ruminant animals caused by the bacterium *Mycobacterium avium* subsp. *paratuberculosis*. Affected animals show diarrhoea, loss of weight, and decreased production performance with consequent economic losses. This bacterium has been detected in some humans suffering from a chronic intestinal disease known as Crohn’s disease (CD) and, therefore, some scientists believe that CD is the human form of paratuberculosis. The disease in small ruminants has been reported in all continents, with goats being more susceptible than sheep. The clinical signs of the disease in goats are not so obvious as often do not show signs of diarrhoea, and the animal may die before being finally diagnosed. In Africa and many developing countries, paratuberculosis is described as a “neglected disease” particularly in small ruminants, which play a vital role in the livelihood of poor communities. This overview attempts to highlight the current research and gaps on this disease in small ruminants to draw more attention for further studies on diagnosis, prevention and control.

**Abstract:**

Paratuberculosis (PTB) is a contagious and chronic enteric disease of ruminants and many non-ruminants caused by *Mycobacterium avium* subsp. *paratuberculosis* (MAP), and is characterised by diarrhoea and progressive emaciation with consequent serious economic losses due to death, early culling, and reduced productivity. In addition, indirect economic losses may arise from trade restrictions. Besides being a production limiting disease, PTB is a potential zoonosis; MAP has been isolated from Crohn’s disease patients and was associated with other human diseases, such as rheumatoid arthritis, Hashimoto’s thyroiditis, Type 1 diabetes, and multiple sclerosis. Paratuberculosis in sheep and goats may be globally distributed though information on the prevalence and economic impact in many developing countries seem to be scanty. Goats are more susceptible to infection than sheep and both species are likely to develop the clinical disease. Ingestion of feed and water contaminated with faeces of MAP-positive animals is the common route of infection, which then spreads horizontally and vertically. In African countries, PTB has been described as a “neglected disease”, and in small ruminants, which support the livelihood of people in rural areas and poor communities, the disease was rarely reported. Prevention and control of small ruminants’ PTB is difficult because diagnostic assays demonstrate poor sensitivity early in the disease process, in addition to the difficulties in identifying subclinically infected animals. Further studies are needed to provide more insight on molecular epidemiology, transmission, and impact on other animals or humans, socio-economic aspects, prevention and control of small ruminant PTB.

## 1. Introduction

Paratuberculosis (PTB) or Johne’s disease (JD) is a chronic contagious disease of animals caused by *Mycobacterium avium* subsp. *paratuberculosis* (MAP). The disease was first described by Johne and Frothington in 1895 and first reported in sheep in Bosnia in 1908 [1]. Paratuberculosis affects mainly domestic and wild ruminants worldwide [2], also, it can affect many non-ruminant animals, such as camels, wild rabbits, pigs, horses, birds, and carnivores [3,4]. Furthermore, MAP has been detected in patients with Crohn’s disease and was associated with other human diseases, such as rheumatoid arthritis, Hashimoto’s thyroiditis, Type 1 diabetes, multiple sclerosis and autism, as presented by Garvey [5]. Thus, the disease can be considered a potential public health hazard [6].

Paratuberculosis can be suspected on clinical signs (intermittent diarrhoea and weight loss despite good appetite) and detection of acid-fast bacilli by microscopic examination in faeces, faecal or tissue culture, serological tests and molecular methods. Undetected subclinical infections greatly contribute to contamination of the environment and spread of the disease [7]. However, one of the main limitations of conducting prevalence studies on PTB is the difficulty in its diagnosis. A suitable, sensitive and confirmatory diagnostic test is a pre-requisite for such studies and hence for effective control programmes [8].

In Africa, PTB can be considered a neglected tropical disease because of little awareness about its occurrence in livestock populations, inadequate documentation and reporting; therefore, it was not considered in research and control programmes [9,10,11]. PTB is an OIE-listed disease (B) and should be reported to the World Organization for Animal Health (OIE) as indicated in the Terrestrial Animal Health Code [12].

The intestinal lesions of PTB cause protein leak, affect the gut microbiome and interfere with gut metabolism causing loss of nutrients and muscle wasting [13,14]. Therefore, as in cattle, PTB in small ruminants causes great economic losses in terms of low weight gains, reduced milk production, early culling and death [15,16], in addition to costs related to diagnosis and disease control [17].

Small ruminants contribute significantly to the alleviation of poverty in poor communities in Africa and Asia through the provision of meat, milk and skins, and as a source of income through animal exports [18]; they are also a compact animal that costs little to feed and do not cost a lot to get their first offspring. This role would be greatly reduced by chronic diseases, such as PTB, in absence of good veterinary services and disease control programmes. However, in countries where sheep and goat farming are well established, production losses due to MAP infection seem to be better documented and economically evaluated [19]. In Australia, the average annual mortality rate due to PTB in 12 sheep flocks was reported to vary between 6.2 and 7.8%, resulting in a 6.4 and 8.5% decrease of the average gross margin [20]. In some flocks, annual mortality approached 20% [21]. In New Zealand, annual losses estimates were US$ 1.5 per MAP infected ewe of fine wool Merino-mainly due to 1.2–2.7% case fatality rate [19]. In the British sheep industry, the annual losses due to PTB were estimated in British Pounds from 0.5 to 16.5 million [22]. It was also reported that dairy sheep and goat farms in Italy suffered a decrease in profit efficiency from 84% to 64% due to MAP infection [23].

## 2. *Mycobacterium avium* subsp. *Paratuberculosis* (MAP)

*Mycobacterium avium* subsp. *paratuberculosis* is a slow growing, non-motile, aerobic, Gram-positive and acid-fast bacillus. Based on phenotypic characteristics (growth rate and pigmentation), two major strains of MAP have been identified: Type I/III and Type II, or MAP-S (sheep type) and MAP-C (cattle type), respectively [24]. Type I isolates are slow growers and mainly affect ovine hosts, while Type II isolates grow faster than type I and commonly affect cattle, in addition to deer, goats, sheep, and other ruminants [25]. MAP Type III isolates are intermediate growers and designated as a subgroup of the sheep or type strain S [24]. However, Type III strains have been isolated from sheep, goats, cattle and camels [26,27]. With the application of one of the genotyping methods, a third MAP type termed “bison” or “type B” for bison isolates was described [25], but further investigations using whole genome data of different MAP strains identified it as a subtype of type C strains [24]. These strains can be cultured from tissues and faeces of MAP infected animals. Cross transmission of strains between ruminant species can be indicated by typing methods [28]. This may be useful in evaluating the spread of MAP in different ruminant species kept under extensive systems where animal mingling is allowed (like Sudan and other African countries).

## 3. Susceptibility to Infection with MAP

Domestic ruminants (cattle, sheep and goat) are the commonly affected animals with MAP [29]. Susceptibility to MAP infection decreases with age; thus age together with the infecting dose and some other factors contribute to limiting the spread of MAP [30]. It is now known that not all animals exposed to MAP develop JD and some appear to clear the infection spontaneously. It is also suspected that some breeds of ruminants are more resistant to MAP infection than others; Merino sheep are reported to be more susceptible to PTB than Romneys [31]. In India, breeds of farm goats of Uttar Pradesh were found to be better adapted to the Indian Bison Type of MAP than those of farm goats in Rajasthan [32]. The study of Begg et al. [33] provides evidence of potential differential disease susceptibility between sheep breeds exposed to MAP infection. However, when compared, goats and cattle are more susceptible and are likely to develop clinical signs of MAP infection, while sheep are more resistant to the development of the clinical disease [34]. Sheep, goats and calves were found fairly comparable as infection models for MAP, though some differences in host responses to infection exist between them [35].

## 4. Transmission of MAP

MAP is mainly transmitted via ingestion of feed or water contaminated with manure. The infection is most common in young animals through ingestion of contaminated colostrum and milk of infected dams. The organism can also be transmitted from an infected pregnant dam to its foetus through the placenta [36]. A non-infected herd generally becomes exposed through herd expansion or replacement purchases of carrier animals [37].

## 5. Clinical Signs of PTB

Animals are usually infected during the first weeks of life, but they can become infected at any age. The clinical PTB in ovine and caprine is mostly observed in animals 2 to 4 years of age; the signs often occur soon after giving birth [38]. Stress factors may hasten the onset of clinical disease. However, clinical signs in sheep or goats are not a reliable indicator of the presence or absence of MAP infection [39]. Weight loss is the predominant clinical sign in infected sheep and goats. In sheep, the period of weight loss differs from one animal to another. Softening of the faeces or diarrhoea occurs only in 20% of the cases at the end stages of the disease [40]. Hypoproteinaemia with intermandibular oedema has been reported in sheep. Besides hypoproteinaemia, a decrease in serum calcium has been observed in sheep and cattle with clinical PTB [41]. Affected animals may show loss of appetite, dullness and rough coat with alopecia [42]. The classical clinical signs of PTB in goats are similar to those in sheep, but with no evidence of diarrhoea [43]. Sub clinically infected goats intermittently shed MAP in faeces up to one-year post-infection. During clinical disease, the animals become emaciated and develop antibodies against MAP, which are detectable in faeces. Advanced clinical disease is associated with progressive weight loss, fragile skin, poor hair coat, submandibular oedema, dehydration, anaemia, and depression [44].

## 6. Prevalence and Distribution of PTB in Small Ruminants

**Figure 1:**
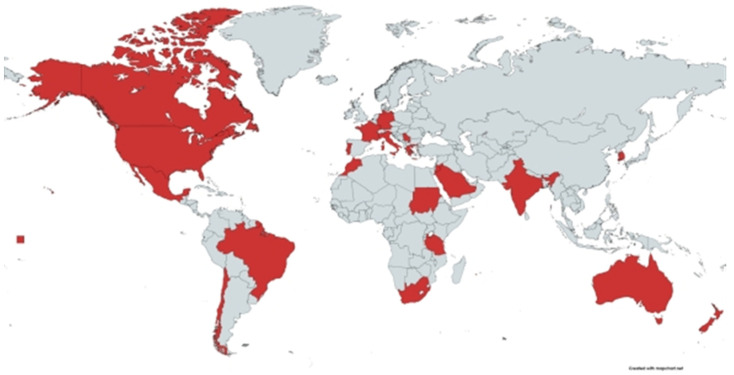
Countries with reported cases of paratuberculosis is small ruminants. The map was created from https://mapchart.net/world.html.

PTB has been reported in many European countries, such as Germany [45], Italy [23] and France [46], as well as in Oceania, Asian and African countries [47,48,49]. In the American continents, caprine PTB was reported in Missouri (USA) [50], Brazil [51], and Canada [52]. In New Zealand, PTB is endemic and widespread in sheep, dairy goats and other animals. Moreover, the disease is found in sheep, goats, dairy and beef cattle, alpaca, llama and deer in the parts of temperate south-eastern Australia [48]. It was noted that no studies have been conducted to provide valid estimates of the prevalence of PTB in sheep and goats in the United States, because of the lack of standardized firm diagnostic tests and funding for research in small ruminants [53]. In the Middle East and Africa, a few reports about PTB in small ruminants have been published. It was reported in sheep and goats in Sudan [54], Morocco [55], Saudi Arabia [56], Jordan [57], South Africa [47] and Egypt [58]. In Figure 1 countries with reported cases are presented; however, it is more likely to be globally distributed.

### 6.1. Prevalence of PTB in Goats at the Animal Level

Prevalence rates of PTB in goats at the animal level vary from country to country and according to the test used. In Quebec (Canada) PTB was diagnosed in 29 out of 152 necropsied goats [52]; a prevalence of 4.3% has been reported in Latin American and Caribbean countries [59]. 17.1% in Eastern Province, Saudi Arabia [56], 7.07% and 15.86% of apparent and true seroprevalence of MAP, respectively in the southwest of Iran [60].

### 6.2. Flock-Level Prevalence of PTB in Goats

The prevalence of PTB in goats at flock-level was recorded in many countries by using ELISA kits for MAP. Prevalence of 14.5% was reported in Italy [23], 83.0% in Grenada, West Indies [61], 0.82% in Chile [62], 1.4% in Missouri, USA [50], 10.9% in Arusha, Northern Tanzania [63], 16.8% in Monteiro, Brazil [51], 3.7% in Latin America and the Caribbean [59], 83% in Ontario, Canada [42], 71% in Germany [45], 3.7%, and 3.9% in Veracruz, Mexico [64,65], and 63.5% in North Gujarat (India) [66]. In the last study, prevalence rates of 28.0%, 7.14% and 12.0%, were obtained for the same animals when screened by Z-N staining, faecal PCR and blood PCR, respectively [66]. In South Korea, the prevalences of 0.8% and 0.6% were obtained using ELISA and faecal culture, respectively [67].

### 6.3. Prevalence of PTB in Sheep at Animal Level

Indirect and conventional tests were used to estimate the prevalence rate of PTB in sheep at the animal level. In Quebec (Canada), 3% prevalence was reported based on characteristic histological lesions in the terminal ileum, ileocecal lymph node and/or ileocecal valve [68]. In the Western and Eastern Cape provinces (South Africa), where the AGID assay was used to identify 52 infected farms, 5% of the sheep population was infected [47]. ELISA test was used in many studies and prevalence rates of 2.3%, 14%, 3.3%, 3.25% and 15.37% in Grenada, Germany, Backa and Srem regions (Serbia), Tunisia and Khuzestan Province of Iran respectively, were reported [45,60,61,69,70]. In Latin America and the Caribbean, the prevalence was 16% [59].

### 6.4. Flock-Level Prevalence of PTB in Sheep

The prevalence rate of PTB using ELISA kits was 3% in each of the Western Cape Province, South Africa and Apulia, southern Italy [23,47], 65% in Germany [45] and 73.7% in Marche region, central Italy [71]. In the northeast of Portugal, a prevalence of 42.7% was detected in 64 flocks by PCR [72]. Valid estimates of the prevalence of MAP infection at the animal and herd levels are important to determine whether the disease warrants interventions to mitigate its negative impact on herd profitability.

Generally, the prevalence of PTB in small ruminants at the animal level estimated by seroprevalence in reporting countries is very low when compared with cattle and buffalo. Moreover, in these large animals, studies that targeted diarrhoeic animals showed a very high seroprevalence of the disease [73]; however, diarrhoea in small ruminants is not a prominent sign of the disease.

As prevalence studies target estimation of the disease either at animal level or herd/flock level or at both, detection of the disease in one animal indicates its occurrence in many others within the herd/flock. On the other hand, prevalence estimates at the herd/flock level are important for knowledge on the disease distribution.

## 7. Pathogenesis of PTB

The pathogenesis of PTB infection in all animals is the same. Neonates and juvenile animals are infected mainly via the oral route from contaminated colostrum and milk.

Transmission may occur by the consumption of milk and colostrum from infected dams [74]. After ingestion, MAP enters the intestinal tract, becomes translocated through the intestinal mucosa mediated by M-cells overlying Peyer’s patches. The bacteria specifically invade the sub-epithelial macrophages, slowly replicate and stimulate the cell mediated immune (CMI) response-initial T cell response [75]. The humoral response is not elicited at the early stages of MAP infection, but later when the CMI response fades and the bacteria are released from macrophages, a strong antibody response is initiated [76]; this usually occurs in advanced clinical cases of PTB. A relationship between immunological responses to MAP and PTB pathology was observed in affected animals, from which two forms were described: the multibacillary and the paucibacillary. In general, the multibacillary (or lepromatous) form is observed when the humoral response becomes predominant and is demonstrated by granulomatous enteritis [77]. This form is more likely to be found in sheep than in other animal species [78]. The paucibacillary (or tuberculoid) form is associated with a strong CMI response and characterized by lymphocytic infiltration in the lamina propria, with few or no visible mycobacteria [77,79,80]; this form has been observed in goats [78,81]. Therefore, the animal responses to any diagnostic test will depend upon the stage of the disease.

## 8. Pathologic Changes of PTB

### 8.1. Pathologic Changes of PTB in Sheep

The gross lesions of PTB in sheep involve thickening of the intestines at various locations with multiple degrees of mucosal corrugation, predominantly near the ileocaecal junction [57,82,83,84,85]. Changes in the caecum and colon are less severe than in the terminal ileum [40,86]. Mesenteric and ileocaecal lymph nodes (MLN and ICLN) are enlarged in up to half of cases and are usually oedematous [77,87]. The histopathological characteristics observed in sheep consist of granulomatous enteritis with marked cellular infiltrate composed of epithelioid, lymphocyte, macrophage and giant cells with acid-fast bacilli [85,86,87]. Villous atrophy, necrosis and hyperplasia of Peyer’s patches have been reported by Coelho et al. [87]. Histopathological changes of MLN and ICLN include infiltration of epithelioid cells and macrophages containing acid-fast organisms [82,87]; also, occasionally, giant cells and foci of caseous necrosis can be seen [88].

### 8.2. Pathologic Changes of PTB in Goats

Thickening and folding of the intestinal wall, corrugation, granular mucosa, serous atrophy of fat and thickening of mesenteric lymphatic vessels were seen grossly. Oedematous enlargement and occasionally calcium deposits were seen in the MLN [89,90]. Greig [91] reported the histopathology of PTB in goats at two stages: infiltration of lymphocytes, plasma cells and macrophages in the lamina propria at the early stages of the disease, and in the late severe stages, macrophages and giant cells can be found in the submucosa and muscle layers. Acid-fast bacilli may be seen in significant numbers. Histopathological lesions were classified into four types (I, II, III and IV) by Hailat et al. [57] and Thakur et al. [90], depending on the type and density of cellular infiltrates (lymphocytes, macrophages and epithelioid cells) in the small intestines and MLN. Lesions are considered grade I if a large number of lymphocytes with very few macrophages and epithelioid cells are found. Infiltration of lymphocytes in a lesser amount than in grade I with some macrophages and epithelioid cells (more than in grade I) are considered as grade II. Abundant numbers of epithelioid cells and macrophages with a small number of lymphocytes to form micro-granuloma are considered as grade III. Grade IV is considered when lesions have few lymphocytes and large amount of epithelioid cells with proliferation of Peyer’s patches and formation of micro-granuloma with giant cells [83]. In goats with advanced PTB, granulomatous lesions were also noted in the liver and lungs [92]. Derakhshandeh et al. [89] reported the diffuse multibacillary lesions, characterized by diffuse granulomatous enteritis and lymphadenitis showing large numbers of epithelioid macrophages in the intestinal lamina propria and cortex of lymph nodes. Lymphangitis and lymphangiectasia in the submucosa and caseous necrosis and calcification in lymph nodes were also noticed. 

## 9. Diagnosis of PTB

Diagnosis of paratuberculosis is based on clinical signs, postmortem lesions, and laboratory confirmation that involves tests for direct detection of the bacteria, such as demonstration of MAP in clinical samples by microscopy, MAP isolation by culturing and detection of the DNA of MAP. The indirect tests as diagnostic assays of MAP infection are based on detection of the host immune response to infection, such as delayed-type hypersensitivity (DTH), interferon assay, enzyme-linked immunosorbent assay (ELISA), agar gel immunodiffusion (AGID) and complement fixation test (CFT) [93,94]. Histopathological analysis is considered a conventional method [95]. Due to variation in PTB presentation from affected to infectious, to an infected animal (termed as “target conditions” or “case definitions”) that have been described as standardized diagnostic criteria for clinical intervention [96,97], sensitivity and specificity of diagnostic techniques to confirm these case definitions vary accordingly. However, a screening technique to confirm the stages of PTB in infected animals is lacking [98]. Therefore, the World Organization for Animal Health (OIE) recommended the evaluation of a diagnostic test after a statement of the purpose of the test [99].

### 9.1. Microscopic Examination

Direct microscopy is used as a rapid technique to detect acid-fast bacilli after preparation of faecal samples and staining by the Ziehl Neelsen (ZN) technique [66]. The sensitivity and specificity of ZN staining are low with difficulties in differentiation between MAP and other acid-fast bacilli. In one of the comparative studies that show the low sensitivity of ZN, Kumthekar et al. [61] detected acid-fast bacilli by ZN staining in only 4 out of 12 samples of ELISA-positive small ruminants, indicating low sensitivity of ZN staining. However, ZN staining is the simplest, fastest, and most economical method of diagnosis and can be used for the initial screening of MAP [100]. 

### 9.2. Culture Methods

Diagnosis of PTB by isolating MAP by culture is the “gold standard” which is considered confirmation method [101]. Moreover, isolation of MAP is difficult due to intermittent shedding of the bacteria and the low number of bacilli in faeces and tissues, respectively [102,103]. Furthermore, MAP is a slow growing organism, which requires several weeks to months for growth in laboratory media. However, incubation of samples with antibiotics before culturing to prevent overgrowth by other faster growing bacteria can lead to killing some MAP bacilli in samples with a low level of bacteria. Therefore, MAP culture from faeces and tissue samples is less sensitive compared with molecular methods and histopathology of lesions to confirm the PTB in animals that were diagnosed clinically [87]. Prior to 1998, the available culture media were not appropriate to support the growth and detection of MAP sheep strains. Radiometric culture has been reported as more sensitive than histopathology and solid media when was used to detect MAP infection in sheep, goats and cattle. In liquid and solid media, the egg yolk and mycobactin J are considered essential additives for the growth of ovine strains of MAP [104,105]. Culture of MAP from goats on Löwenstein-Jensen, Herrold’s egg yolk medium (HEYM) with and without sodium pyruvate and Middlebrook 7H11 containing mycobactin J has been used [106]. Goats can be infected by various MAP strains and, therefore, different media and an incubation period of up to 6 months should be expected before getting detectable growth of MAP in culture.

### 9.3. Molecular Assays

Molecular assays are useful techniques in the diagnosis of PTB in suspected animals’ faeces and blood, as they improve the sensitivity of detection of MAP by targeting its genome. However, there is potential for cross-reactions or inhibition from biological substances for these assays. MAP genome in the faecal and blood samples is detected in extracted DNA by PCR amplification of the insertion sequence 900 (IS900) element [66,87]. Sonawane and Tripathi [107] found 251 gene PCR is better than IS900 in the detection of MAP from the tissues. Additionally, PCR was found to be more sensitive than a histopathological examination of 66 suspected goat carcasses with PTB [89], while nine (13.63%) carcasses were positive for MAP in both histopathology and PCR, eight were positive in PCR without histopathological lesions related to PTB. The insertion sequence 1311 (IS1311) has also been used in nested PCR to amplify the MAP DNA of caprine tissue isolates [108]. Multiplex PCR based on the IS900, IS901, IS1245 and the dnaJ gene has been developed to overcome false-positive results arising from the presence of IS900-like insertion sequences in other mycobacteria. However, because of reagent interference and primer dimers, the sensitivity of this test is still low [94,109]. A more sensitive and specific real-time PCR assay was developed for detecting MAP, based on the combination of IS900 and 251 genomic loci, which was identified as MAP-specific with a set of specific primers and probe, as described by Rajeev et al. [110]. In other studies, an F57-based real-time PCR system was used to detect MAP in milk or cheeses [111,112]. Moreover, a loop-mediated isothermal amplification assay (LAMP) targeting *ISMap02* was used as a rapid and sensitive detection tool for MAP in small ruminants [113].

### 9.4. Serologic Tests

The serologic tests used as diagnostic techniques for PTB in small ruminants include AGID, CFT and the ELISA. These tests are very important in small ruminants, in which the culture of faeces has low sensitivity and is costly [114]. Goats, in comparison with sheep, have strong and early antibody responses suggesting that current serological tests may be more sensitive in this species [115].

#### 9.4.1. Enzyme-Linked Immunosorbent Assays (ELSIA)

The sensitivities and the specificities of ELISA assays to detect PTB in small ruminants are in the range 16–100% and 79–100%, respectively. Therefore, the variations in the sensitivity and specificity of ELISA assays should be interpreted with attention [97], despite these variations, the ELISA has been used in domestic animals as a screening test [116]. However, an indigenous ELISA kit was found superior to commercial ELISA kits in the detection of PTB in sheep and goats in India [117]. Moreover, milk ELISAs for PTB in goats, relative to faecal culture was found to be a cost-effective and accurate alternative [62]. Additionally, ELISA has been proven useful for the detection of ovine PTB with estimated specificity of 98.2 to 99.5% and sensitivity of 35 to 54% [118].

#### 9.4.2. Agar Gel Immuno-Diffusion (AGID) Test

The AGID has been reported as a successful screening method in control programmes of PTB in cattle, sheep and goats [100]. In earlier studies, its specificity was reported as 100%. Moreover, the test showed higher sensitivity and specificity than ELISAs when it was used in small ruminants in New Zealand and Australia [118,119,120] and was reported as better than the absorbed ELISA in detecting MAP-infected sheep with poor body condition [118]. However, in later reports, the sensitivity of AGID was found to be less than that of the ELISA [121,122]. The specificity and sensitivity of AGID measured against ELISA were 99% to 100% (95% CI) and 38% to 56% (95% CI), respectively [118]. Kumthekar et al. [61] found that out of 12 ELISA-positive small ruminants, only five animals were positive when they were tested by a commercial AGID assay. 

#### 9.4.3. The Complement Fixation Test (CFT)

CFT is used for the screening of PTB in suspected animals [123,124]. The sensitivity of the CFT has been reported in a range of 10 to 90% [125,126,127]. The specificity of CFT was less than AGID and ELISA as reported by Singh et al. [128]. However, in Japan, the CFT is requested by importing countries and is used for diagnosis in small ruminants (sheep and goats) combined with a Johnin skin test [19]. Moreover, the confirmation of the clinical diagnosis of PTB by CFT is recommended in Europe, although it is considered less accurate than the ELISA with respect to sensitivity and specificity [129]. Diagnostic techniques used for PTB in small ruminants from 2012 to 2020 are summarized in Table 1.

## 10. Treatment, Control and Prevention

Successful treatment of PTB has not been reported in infected animals [124]; however, control programmes for dairy cattle can be applied to dairy goats and sheep. Changes in management practices in order to reduce the transmission of MAP as well as the test-and-cull method to eliminate shedding of MAP and using vaccination to increase resistance to infection, all these methods had been reported as the main approaches to control and eradicate PTB [19,133,134]. In addition, biosecurity is the essential approach in uninfected animals for reducing both within-farm and between-farms spread of infection [94].

### 10.1. Changes of Management Practices

To cut off the transmission of MAP, good management practices are an important approach for controlling ovine/caprine PTB, especially in small flocks/herds. These management practices involve feeding uncontaminated colostrum and milk replacement products, rearing young stock separately from the adults, separating offspring from dams, minimizing the contact between infected adult goats, sheep and others, avoiding exposure to potentially infected adult animals, their manure and the contaminated environment were recognized as control measures of PTB within-farms [42,135,136]. Producer knowledge, diligence and investments have been reported as essential elements in the effectiveness of this approach through improving the biosecurity practices [137].

### 10.2. Test-and-Cull

The effectiveness and repetition of diagnostic techniques are considered the main issue for test and cull strategies to identify the early infection of MAP in animals, particularly before their incipience of faecal shedding [138]. Therefore, the limited application of this strategy in sheep and goats is attributed to relation between the individual value of animals and the high cost of diagnostic tests with variations in their sensitivities [137]. Moreover, diagnostic tests are critical issues in control programmes of PTB. As the time-interval between the infections and the animal shows clinical signs and/or gives positive results in diagnostics tests is very long, the test and cull approach would be difficult [134]. However, the combination of vaccination with ‘test and cull’ was found to be economical as well as a more effective strategy to control PTB in various herds of goats, buffaloes and cattle [139,140].

### 10.3. Vaccination

Paratuberculosis vaccine is commonly applied in small ruminants to reduce the clinical disease because vaccines reduce the shedding of MAP by infected animals and lower the severity of clinical cases [53,141]. Vaccination is cost-effective strategy compared with other control strategies [134,142,143]. Many countries have applied the strategy of vaccination for sheep successfully [133,142]. However, vaccination is not considered the best option as a control measure and is even prohibited in some countries because of interference with the skin test for diagnosis of tuberculosis. Luckily, new promising approaches to overcome this interference have been applied successfully by using proteinic and peptidic cocktails in skin tests instead of traditional test reagents [144]. On the other hand, in a number of countries, such as Australia [145], New Zealand [146], Spain [147], India [148] and The Netherlands [149], vaccination as a management measure to control paratuberculosis has been used.

It is recommended that vaccination of small ruminants against PTB be done in very young animals to prevent interference with the diagnosis of tuberculosis. Vaccination trials in Australian sheep indicated 8 months as the age threshold for vaccination efficacy [150]. Persistence of antibodies for up to 42 months post-vaccination was reported, but infection from the environment could not be ruled out to have a booster effect leading to this long persistence [151]. Dairy goats in infected herds in The Netherlands are commonly vaccinated once during the first months of life [149].

Currently, the vaccines in use against PTB include live (non-attenuated and attenuated) and killed whole cell vaccines, as well as subunit vaccines which have been used in a few cases with less degree of protection [134,152,153]. Based on efficacy, both the inactivated (killed) vaccines and attenuated vaccines were equally effective [154]. However, many countries do not prefer live vaccines because of the partial protection that might be provided by reducing the clinical cases, not the eradication of infection with frequently diminishing immunity of vaccinated animals when are sold to other herds; also, perhaps because of public health issue by infecting humans [134].

### 10.4. Selective Breeding

Evidence of breed susceptibility to PTB has been reported as mentioned earlier in this review. Therefore, the role of host genetics can be an alternative approach to control chronic diseases like JD [155,156]. Breeding for disease resistance would be an effective means for controlling PTB in domestic ruminants.

## 11. Research Gaps

Effects of PTB of small ruminants on other animals or humans and its socioeconomics received no attention; molecular epidemiology of MAP and its dynamics of transmission, in addition to the role of the gut microbiome in susceptibility and resistance to MAP infection have not been yet addressed in small ruminants. Studies evaluating strategies for controlling PTB in small ruminants, such as test-and-cull and/or vaccination are scanty and limited; and evaluation of pooled samples in screening the disease with different diagnostic tests is yet to be addressed.

## 12. Conclusions

A few studies about PTB in small ruminants have been published, especially from Africa and the Middle East. Therefore, prevention and control programmes for PTB in small ruminants have not been established in many countries. Further studies investigating the prevalence of PTB in small ruminants can provide important insights into setup the first step in prevention and control. Increasing public awareness about the possible effects of MAP on human health requires intensive work. 

Small ruminants, especially goats, in developing countries play an important role in maintaining the livelihood and food security of people in rural areas, in addition to their contribution to the national economies of many countries. Difficulties in identifying subclinical cases and limitations of available diagnostics, combined with the negligence of the disease make PTB in small ruminants a hidden killer in most cases.

## Figures and Tables

**Table 1:** Diagnosis of paratuberculosis in small ruminants using different techniques (2012–2020).

No. of Studies	Total No. of Animals or Samples	Smears from Faeces or Tissue/Inclusion Criteria	PCR/Inclusion Criteria	Real-Time PCR	ELISA	AGID/Inclusion Criteria	Culture	Histopathology	Ref.
1	479 sheep 260 goats	4/5 AGID +ve			11 (2.3%) sheep 1 (0%) goat	5/12 ELISA +ve		2 sheep +ve in 3 tests	[61]
2	219 goats	9.2% (7/76)	12.5% (5/40)		43.3% (95/219)	10% (24/219)			[130]
3	200 sera from goats	14/50(28.0%) (strong reactors in ELISA)	1/14 (7.14%) +ve faecal smears and 6/50 (12.0%) strong ELISA +ve.		63.5%				[66]
4	30 sheep		4 (13.3%) faeces, 19 (63.3%) tissues, 7 (23.3%) blood		3 (10.0%)		2 (6.7%) faeces, 6 (20.0%) tissues	21 (70.0%)	[87]
5	66 slaughtered goats		9 (13.63%) tissue					9 (13.63%)	[89]
6	130 (8.7%) suspected small ruminants	62 (47.7%) faeces		25 (65.8%)/38 +ve faecal smears					[85]
7	192 goats				21 (10.9%; 7.3–16.1%)				[63]
8	168 sheep (farm 1), 112 sheep (farm 2)	30 (60.0%), 5 (10.0%)	24 (35.2%) 6 (50.0%)		38 (76.0%) 7 (14.0%)				[131]
9	121 serum samples 16 pooled faecal samples	2/16 faecal samples	11/23 (9%) ELISA strong +ve			23/121 (19.01%)/strong +ve 85/121 (70.25%) +ve			[132]
